# Role of ELISPOT Assays in Risk Assessment Pre- and Post-Kidney Transplantation

**DOI:** 10.3390/cells1020100

**Published:** 2012-05-10

**Authors:** Jennifer R. Zitzner, Anat R. Tambur

**Affiliations:** Comprehensive Transplant Center Feinberg School of Medicine, Northwestern University, 303 E Chicago Ave., Tarry Building Suite 11-703, Chicago, IL 60611-3008, USA; E-Mail: a-tambur@northwestern.edu

**Keywords:** ELISPOT, kidney, transplant, risk assessment

## Abstract

Immunologic risk in kidney transplantation is typically minimized by avoiding, or at least limiting, the potential of donor specific humoral responses by testing for the presence of donor-specific antibodies (DSA). Additionally, selecting donor and recipient pairs with the least number of human leukocyte antigen (HLA) mismatches has been shown to play a role in transplant outcome. However, numerous other factors may play a role in the success of transplant outcome and patient health. Specifically, the use of T-cell allospecific ELISPOT assays have helped elucidate the role of pre-formed cellular responses as additional factors in post-transplant outcome. In this review, we will evaluate numerous uses of ELISPOT assays to assess the pre- and post-transplant immunologic risk of rejection episodes, graft survival and even viral susceptibility as well as the utility of ELISPOT assays in monitoring tolerance and withdrawal of immunosuppressive medications following kidney transplantation.

## 1. Introduction

Risk assessment in transplantation is essential in order to determine the most appropriate donor/recipient pair and minimize the occurrence of rejection. Strategies to evaluate risk are also used when determining appropriate immunosuppression methods pre- and post-transplant to ensure adequate protection against rejection while minimizing complications including drug toxicity, viral infections and malignancies. Risk stratification is also useful in determining the frequency of post-transplant follow-up visits and potential immunosuppression withdrawal. Risk and immune status pre- and post-transplant can be assessed in several ways. 

Pre-transplant sensitization can occur as a result of previous transplants, transfusions, or pregnancies. Recipient humoral sensitization status is determined by analyzing recipients’ serum for the presence of pre-formed HLA antibodies using tests such as the panel reactive antibody (PRA) test and more recently solid phase single antigen testing. Cellular sensitization, although not routinely examined, focuses on the reactivity of cellular components—mainly T cells. Similar to the PRA test for serum antibodies, the panel reactive T cell (PRT) test has been developed to determine the frequency of interferon (IFN)-gamma producing memory T cells against a panel of allogeneic cells [1,2,3]. Similar to PRA testing, the PRT test provides a measure of recipient sensitization, but neither is donor specific. 

Several groups have used microarrays to determine protein or microRNA “signatures” in blood and urine as a means to assess post-transplant risk of poor graft outcome [4,5,6,7,8]. Markers in the blood and urine such as B-cell activating factor (BAFF) [9,10] and proteinuria [11,12,13,14] have been associated with renal graft outcome. Additionally, stimulation-dependent adenosine triphosphate (ATP) release from CD4T+ cells (analyzed using the ImmuKnow assay (Cylex, Inc. Columbia, MD) have been examined as a marker of increased T cell immune activation. However, studies using the ImmuKnow assay to correlate pre- and post-transplant T-cell immune activation status with graft outcome have had conflicting results [15,16,17,18,19]. Similarly, the presence of soluble CD30, a membrane-bound molecule proteolytically cleaved in activated T cells, has been proposed as a biomarker for increased risk of kidney and lung graft rejection, but has also been met with conflicting results [20,21,22,23,24]. 

The previously mentioned methods to assess risk in kidney transplant recipients can be influenced by non-donor-specific factors. Donor-specific methods of evaluating pre- and post-transplant risk are believed to provide a more accurate assessment of transplant outcome. Protocol biopsies allow for direct visualization of kidney pathology and characterization of cellular infiltrates, but are limited by sampling error, invasiveness, associated risks, and cost. Solid phase single antigen beads allow testing for the presence and semi-quantitative strength of DSA pre- and post-transplant. Donor-specific cellular sensitization and immunity were previously assessed using mixed lymphocyte reactions (MLR), in which recipient cells were co-cultured with donor irradiated cells and proliferation was measured 5–7 days later. However, MLR assays proved to be have significant false positive as well as false negative results and therefore had little predictive value of transplant outcome [25,26,27]. 

A more sensitive modification of MLR is the ELISPOT assay. The ELISPOT method, like the MLR, has the advantage that it is a functional assay that can detect the ability of naïve or memory cells to respond to a target by monitoring cytokines produced by the responder cells. Memory cells are cells that have previously seen a particular antigen, have less stringent requirements for re-activation and therefore can secrete a wide range of cytokines in response to stimulation more rapidly (within 24 h) than naïve cells. While not routine in many transplant centers, donor-specific T cell immunity pre-and post-transplant is an area of active research. Recipient T cell reactivity to donor, viral or bacterial antigens can be evaluated to determine risk of rejection episodes or infections pre- and post-transplant. The information obtained from these assays may also help in determining individualized immunosuppressive therapy regimens as well as tapering or complete withdrawal of immunosuppression in cases where risk is low. In this review, we will highlight the current research and utility of IFN-gamma ELISPOT assays in risk assessment pre- and post- kidney transplantation.

## 2. Results and Discussion

### 2.1. Pre-Transplant Donor T Cell Reactivity and Early Acute Rejection Episodes

ELISPOT assays can detect a range of cytokines produced by cells. We will focus in this review specifically on IFN-gamma produced by memory T cells, as it has been shown to be associated with increased risk of rejection episodes. While patients with a positive PRT prior to transplant have been shown to correlate with acute rejection [3,28], similar to PRA, the results from PRT only provide an overall risk stratification factor. These tests do not provide the necessary and more precise risk stratification that is achieved by assessing donor-specific responses. For example, while high PRA is indeed associated with statistically increased risk, the more precise risk stratification is achieved by assessing donor-specific antibodies. Similarly, memory response by IFN-gamma production should be assessed in a donor-specific manner. Several additional studies have been aimed at determining whether donor-specific T cell reactivity is an indicator of acute rejection episodes post-transplant. 

Koscielska-Kasprzak *et al*. retrospectively studied pre-transplant donor-specific T cell reactivity of 53 renal transplant recipients using the IFN-gamma ELISPOT assay [29]. Higher donor-specific T cell reactivity as indicated by an increased spot number (*p* < 0.05), median spot size (*p* < 0.05), and intensity (*p* < 0.05) was found in patients who experienced a biopsy-proven rejection episode within the first year after transplant. Fourteen of the 16 patients experiencing an acute rejection episode had a positive ELISPOT result compared with only one of the 16 patients that had elevated PRA alone, suggesting that the predictive power of the donor-specific ELISPOT was greater than PRA status.

Kim *et al*. studied the correlation of pre-transplant IFN-gamma frequencies with clinical outcome at 6 months post-transplant in 45 living donor renal transplant recipients [30]. During the first 6 months post-transplant, 11 patients developed an acute rejection episode. The mean frequency of donor-specific IFN-gamma spots was significantly greater in patients who experienced acute rejection episodes in the first six months following transplant (*p* < 0.001). The IFN-gamma ELISPOT assay identified patients that later developed acute rejection episodes with a sensitivity of 81.8% and a specificity of 64.7%. Positive pre-transplant ELISPOT results also correlated with increased serum creatinine and lower glomerular filtration rate at 6 months post-transplant. As with the previous study, the authors found no correlation between recipient PRA and acute rejection episodes.

Not all studies have found a positive correlation between pre-transplant positive ELISPOT results and acute rejection episodes. Reinsmoen *et al*. [31] analyzed 126 kidney transplant recipients in a three-arm “steroid-sparing” immunosuppressive treatment study. Given that no significant differences were seen in patient demographics or outcomes in the three patient populations studied, results from all three groups were analyzed together. Donor-specific IFN-gamma T cell precursor frequencies were compared with several clinical outcomes including acute rejection episodes. An increase in pre-transplant donor-specific IFN-gamma ELISPOT results was not associated with early (<3 months) or late (>3 months) acute rejection episodes post-transplant. 

Although the conflicting results presented may seem to indicate that the IFN-gamma ELISPOT assay is not an ideal measurement of acute rejection risk post-transplant, the clinical transplant protocol in each study differs. Unlike the Koscielska-Kasprzak and Kim studies, the recipients in the Reinsmoen study received induction therapy. Induction therapy aims to reduce the number of circulating T cells to prevent early acute rejection episodes. As seen in their study and described further in the following section, induction therapies can selectively deplete subsets of T cells. The negative correlation of pre-transplant ELISPOT results and acute rejection seen in the Reinsmoen study may be due to a selective reduction in donor-specific IFN-gamma producing T cells. Therefore, the contrasting results between the studies may be due to protocol-specific factors. It also indicates that the predictive power of the IFN-gamma ELISPOT assay may be center and protocol dependent and may not be useful in centers that use induction therapy. 

Additionally, the first two studies described noted no correlation between rejection episodes and pre-transplant PRA and a partial conclusion was drawn that ELISPOT results were a greater predictor of acute rejection than PRA. However, it is important to highlight that the patients chosen for both of these studies had negative flow cytometric crossmatches, indicating that there were little or no donor-specific antibodies present at the time of transplant. In the final study described, patients with HLA-specific (determined by a positive cytotoxic crossmatch) and donor-specific antibodies by solid phase single antigen analysis were at increased risk for acute rejection. 

### 2.2. Risk assessment in the Choice of Induction Therapy

Induction therapy is used to deplete or inhibit preformed memory T cells that can negatively impact the renal transplant. However, suppression of T cells from induction therapies can leave the recipient vulnerable to malignancies and infections. Therefore, it is desirable to limit the severity of induction therapy used while keeping the risk of rejection episodes low. Induction therapies work through different mechanisms. Antithymocyte globulin (ATG) works though depleting T cells, whereas other therapies affect cellular alloreactivity by blocking T cells (IL-2 receptor blockers), or by depleting all mononuclear cells expressing CD52 (Alemtuzumab). Depleting therapies are believed to be more effective in higher risk patients while blocking therapies may be sufficient in lower risk patients. The impact of induction therapies on T cell depletion and rejection episodes has been the focus of multiple studies, two of which are outlined below.

In a retrospective analysis of 130 kidney (n = 119) and kidney/pancreas (n = 11) transplant recipients, Augustine *et al*. examined the power of pre-transplant ELISPOT assays in predicting rejection episodes within the first 12 months following transplant [32]. Patients were differentiated by those who received induction therapy, T cell depletion (ATG) or IL-2 receptor blockers (basiliximab), and those who did not receive induction therapy pre-transplant. Of the 32 patients who had positive ELISPOT assays, those with induction therapy showed no rejection episodes whereas 46% of those without induction therapy experienced an acute rejection episode (*p* = 0.02). Among the ELISPOT negative group, acute rejection episodes were similar regardless of the use of induction therapy. This group continued to analyze post-transplant IFN-gamma ELISPOT results. Their data revealed that within the first six months following transplant, six of seven ELISPOT positive patients with induction therapy converted to an ELISPOT negative status. However, in ELISPOT positive patients who did not receive induction therapy, only six of 17 converted to an ELISPOT negative status. The mechanism of conversion was not explored. 

Using the IFN-gamma ELISPOT assay, Cherkassky *et al*. prospectively studied 31 kidney transplant recipients to determine whether induction therapy with ATG or IL-2 receptor blockers had a differential effect on donor-reactive T cells [33]. ATG induction therapy reduced the number of T cells although the results were more pronounced in the CD4 T cell population while IL-2 receptor blockers had a minimal effect on T cell numbers. Additionally, after adjusting for T cell numbers, ATG induction therapy resulted in a greater effect on alloreactive T cells 3 months after transplant compared to patients with IL-2 receptor blocker treatments. Donor hyporesponsiveness was still seen at 6 months for the ATG group but not in the IL-2 receptor blocker group. 

These studies highlight that IFN-gamma ELISPOT assays may have a utility in stratifying patients into high-risk and low risk categories. Patients with lower pre-transplant cellular reactivity may be candidates for a lower risk induction therapy method while those with high cellular alloreactivity may require a stronger induction therapy in order to prevent acute rejection episodes. Additionally, results from Augustine *et al*. suggest that while patients with donor-specific alloreactive T cells may benefit from induction therapy, patients without pre-transplant cellular alloimmunity may not need induction therapy at all. 

### 2.3. IFN-Gamma ELISPOT as a Tool to Monitor Viral-Specific T Cell Recovery

There is a delicate balance between a level of immunosuppression to prevent rejection episodes and oversuppression of the immune system leaving the patient susceptible to infections. Predicting the risk of patient susceptibility to viral strains as well as monitoring recovery of antigen-specific viral T cells is helpful in minimizing the amount and determining the most appropriate type of immunosuppression for an individual. Several opportunistic infections are seen in renal transplant recipients and include cytomegalovirus (CMV), polyomavirus BK, and in some populations, tuberculosis (TB). The risk of acquiring or reactivation of each of these viruses is dependent on the recipient and donor status pre-transplant as well as immunosuppression status post-transplant. Using IFN-gamma ELISPOT assays, several studies have been aimed at monitoring the recovery of viral-specific T cells post-transplant and the impact of various types of immunosuppression on each viral strain. 

Abate *et al*. examined 85 kidney transplant recipients (70 CMV positive, 15 CMV negative) to determine the rate and uniformity of CMV-specific T cell recovery after transplantation [34]. In CMV positive recipients, there was a marked decrease in CMV-specific T cells as a result of immunosuppression after transplantation followed by a steady and constant reconstitution from day 60 to 360. However, the rate of reconstitution for each individual patient varied. CMV negative recipients, regardless of donor serostatus, did not develop a CMV-specific T cell response while on prophylaxis treatment (180 days following transplant) and only developed T cell immunity after an episode of CMV viremia. While pre-transplant CVM serostatus and antiviral therapies influenced T cell reconstitution, ATG induction therapy was not found to significantly impact the reconstitution of CMV-specific T cells. 

Egli *et al*. evaluated the effect of immunosuppressive doses on polyomavirus BK specific T cells in 16 kidney transplant recipients receiving tacrolimus, mycophenolic acid and prednisone who had detectable levels of BK virus in their urine within the last 6 months [35]. The mean time between detection of BK virus in the urine, a decrease in immunosuppression, and ELISPOT analysis was 4 weeks. The numbers of IFN-gamma producing polyomavirus BK specific T cells were inversely correlated to levels of tacrolimus *in vivo*, but not mycophenolate or prednisone post-transplant. In another set of experiments, cells from 14 CMV and BK virus seropositive healthy donors were cultured *in vitro* with varying doses of immunosuppressive treatments. IFN-gamma ELISPOT results examining BK virus-specific T cells showed a dose-dependent inhibition of viral-specific T cells for tacrolimus and cyclosporine, but not sirolimus. 

When current clinical tests cannot accurately predict the risk of developing a viral disease, IFN-gamma analysis of viral-specific T cells may provide insight to patients at a higher risk. Kim *et al*. studied a South Korean population of 312 kidney transplant recipients (40 TB positive, 272 TB negative) to analyze the predictive power of the IFN-gamma ELISPOT assay in detecting patients at risk for developing TB [36]. Patients with a pre-transplant positive skin test or clinical risk factors for TB were treated with isoniazid and did not develop TB. However, in the patient population with no clinical symptoms or positive skin test for TB pre-transplant, positive TB-specific ELISPOT results pre-transplant were able to predict a risk of TB infection following transplantation whereas none of the patients with a negative TB-specific ELISPOT assay developed TB (*p* < 0.001). 

The previous studies indicate that pre- and post-transplant viral specific ELISPOT assays can be effective in determining risk of developing viral infections post-transplant as well as help to individualize immunosuppressive treatments by identifying patients with viral-specific T-cell recovery. These assays also may highlight patients who either need to have immunosuppression doses lowered or are at risk of acquiring long-term viral infections. While the *in vitro* studies from Egli *et al*. cannot be directly translated into clinical practice, their studies suggest that patients at a higher risk of developing viral infections may benefit from alternative immunosuppressive therapies.

### 2.4. IFN-Gamma ELISPOT as a Tool to Predict Candidates for Immunosuppressive Therapy Withdrawal

Advances in immunosuppressive therapy have significantly reduced the incidence of acute rejection and increased short-term graft survival. However, long-term maintenance of immunosuppression regimens can lead to drug toxicities, infections, and malignancies. Therefore, many centers are working to identify protocols that can induce tolerance of the transplanted kidney in order to minimize or eliminate the need for immunosuppression. One limitation of these protocols is identifying the subset of patients who have developed tolerance, as currently there are no reliable assays to evaluate when a patient has reached tolerance. Recent studies have been aimed at determining immune profiles of patients who are operationally tolerant (stable after withdrawal from immunosuppressive drugs), and therefore may be able to be weaned off of immunosuppression.

Donor bone marrow cell (DBMC) infusions prior to renal transplant have been associated with operational tolerance. Matthew *et al*. studied three groups of transplant recipients using *ex vivo* methods to identify recipients with immune profiles conducive for immunosuppressive therapy withdrawal [37]. The groups consisted of DBMC-infused haploidentical recipients (n = 20), control haploidentical recipients (n = 8), and HLA identical recipients (n = 11). All recipients studied were on immunosuppressive regimens throughout the time of the study. Results showed that most (11 of 17) DBMC infused recipients had negative donor-specific IFN-gamma ELISPOT assays. Similar results were seen in the two remaining groups. Each group showed lower donor-specific responses as compared to third party responses. Recipients with positive IFN-gamma ELISPOTS were often donor-reactive in other assays monitored.

In a multicenter European study, Sagoo *et al*. examined the immune profiles of 71 kidney transplant recipients and 19 age-and sex-matched healthy controls using several biomarkers and bioassays [38]. The kidney transplant recipient group was comprised of 3 subgroups: operationally tolerant recipients (n = 11), recipients on various immunosuppressive regimens (n = 51), and recipients undergoing chronic rejection (n = 9). The majority of transplants were from cadaveric donors (7 of 11) and highly HLA mismatched (median number of mismatches, 4.0). Among the several immune parameters tested, the ratio of IFN-gamma ELISPOT responses to donor stimulator cells and third party (but HLA donor matched) cells revealed a donor-specific hyporesponsiveness in operationally tolerant recipients as compared to stable recipients on immunosuppression. Donor-specific hyporesponsiveness was not mediated by T regulatory cells as the ratio did not change when CD4+CD25+ responder cells were depleted. To validate their results, the same parameters were tested in the Immune Tolerance Network (ITN) cohort of kidney transplant recipients that included 24 operationally tolerant transplant recipients. The ITN cohort consisted of highly HLA matched (median number of mismatches, 0.0) living donor kidney recipients. Although the same trend appeared in this cohort, both anti-donor and anti-third party responses were low and no significant results were reached between any of the groups.

The differences in correlation of IFN-gamma ELISPOT assays and operationally tolerant recipients (or recipients under an operational tolerance protocol) highlight the importance of each of the populations studied. Matthew *et al*. and the ITN study cohort did not show significant differences in donor specific ELISPOT responses of recipients under an operational tolerance protocol or operationally tolerant populations, respectively, whereas, the European cohort showed significantly lower donor-specific responses in operationally tolerant recipients. The differences shown may lie in the degree of HLA matching of the donor and recipient as well as living *versus* cadaveric donors. Additionally, it is important to note that the IFN-gamma ELISPOT assay was used as a component in each of these studies. While T cell donor hyporesponsiveness is a component of a tolerant immune profile, results offered one piece of a larger network of immune responses.

## 3. Conclusions

Risk assessment in kidney transplantation is complex and dependent on multiple factors. While rejection can usually be minimized by the use of immunosuppressive therapies, it can be at the expense of increased risks due to drug toxicity and susceptibility to infections and malignancies. Transplant clinicians are often required to arbitrarily adjust medications to counter effects of other immunologic factors. Many of these changes are done empirically, with no solid evidence based data. The introduction of the IFN-gamma ELISPOT assays may provide a tool to predict and monitor pre- and post-transplant risk factors in order to individualize immunosuppressive therapies.

While the IFN-gamma ELISPOT assay can be utilized to look at cellular reactivity in transplant recipients, it is important to underscore that results from this assay assess only one aspect of the immune system. The addition of this assay to, but not replacement of, current risk assessment strategies such as protocol biopsies and monitoring of DSA will strengthen the understanding of individual risk management. Patients can further be stratified into higher and lower risk categories so that immunosuppressive therapies can be tailored optimally to a particular patient. 

While the studies correlated clinical outcomes to ELISPOT assay results, often there were only single time points analyzed. This strategy is useful when matching transplant donors and recipients pre-transplant. However, to get a broad picture of recipient risk and how to most effectively manage patient therapy post-transplant, analysis of serial time points may offer a better analysis of recipient risk status. Further studies are also needed to more fully understand the benefit of IFN-gamma ELISPOT assays in the prediction of long-term clinical outcomes. Moreover, many of the current studies were all done in a retrospective fashion. It is critical to further evaluate the positive predictive value as well as the negative predictive value of these assays in a prospective manner. 

Finally, the utility of the ELISPOT assay as a method for transplant risk must be evaluated on an individual center basis. As seen across studies with conflicting results, the predictive power of the assay depends on several variables such as the induction protocol used or the level of HLA matching. It is therefore important to have each center evaluate the predictability of the IFN-gamma ELISPOT assay specifically for their patient population. 
